# Investigating Affective Responses to Remotely Delivered “At Home” Low Volume High Intensity Interval Exercise: A Non-Randomized Parallel Group Feasibility Study

**DOI:** 10.3389/fspor.2022.862019

**Published:** 2022-07-06

**Authors:** Imogen Howard, Ailsa Niven, Paul Kelly, Shaun M. Phillips

**Affiliations:** ^1^Institute for Sport, Physical Education and Health Sciences, The University of Edinburgh, Edinburgh, United Kingdom; ^2^Physical Activity for Health Research Centre, The University of Edinburgh, Edinburgh, United Kingdom; ^3^Human Performance Science Research Group, The University of Edinburgh, Edinburgh, United Kingdom

**Keywords:** tolerance, LV-HIIE, circuit training, adherence, affect

## Abstract

**Background:**

Low volume-high intensity interval exercise (LV-HIIE) has gained interest, due to its efficiency in invoking health and fitness benefits. However, little research has studied “at home” feasibility or effects of LV-HIIE. This study aimed to demonstrate that remote “at-home” LV-HIIE research is possible and to investigate if affective responses to the LV-HIIE protocol, subsequent intentions, and self-efficacy to repeat were related to self-reported tolerance of the intensity of exercise.

**Methods:**

Using self-reported tolerance of the intensity of exercise, 41 healthy, physically active participants (25 female and 16 male; age 21.3 ± 1.0 years, body mass index 23.0 ± 2.9 kg.m^2^) were divided into low tolerance (LT, *n* = 14), middle tolerance (MT, *n* = 15), and high tolerance (HT, *n* = 12) groups. Participants completed a 20-min LV-HIIE circuit training video [2 × (10 ×30 s work, 15 s rest)] at home. Participants reported ratings of perceived exertion, affective valence, and perceived activation at baseline, during the protocol, immediately post-protocol, and during the cool down. 20-min after completion, respondents answered questions on exercise task self-efficacy and intentions to repeat LV-HIIE.

**Results:**

The study recruited *n* = 65 individuals, of whom *n* = 50 passed screening. Ultimately *n* = 41 (82%) completed the exercise protocol and data collection. Ratings of perceived exertion were not significantly different between groups (*p* = 0.56), indicating similar perceptions of task difficulty. There was no significant effect of tolerance on affective valence (*p* = 0.36) or felt arousal (*p* = 0.06). There was evidence of high individual variability in affective responses within and between participants. Subsequent intentions and self-efficacy to repeat the exercise protocol did not seem to be related to affective valence during or after the protocol.

**Discussion:**

Recruitment and data collection indicated that research into “at home” LV-HIIE is possible. High individual differences in affective responses suggest that LV-HEII may be appropriate for some but not all as an exercise option. Assessing self-reported tolerance of intensity of exercise may not appropriately identify whether or not LV-HIIE will be suitable for an individual.

## Introduction

Despite the well-established physical and mental health benefits of physical activity (Lear et al., 2017; Biddle et al., 2021), it is estimated that globally one in four adults do not reach the recommended levels of physical activity (Guthold et al., 2018). Although the determinants of physical activity are likely multi-factorial, perceived lack of time is a commonly cited reason for being inactive (Hallal et al., 2012). This barrier of perceived lack of time has helped fuel a growth in the promotion of high-intensity interval exercise (HIIE), which may provide equivalent health benefits to more traditional exercise, but with less time commitment.

HIIE has been defined as brief repeated bursts of relatively intense or all-out exercise separated by rest or low-intensity exercise (Gillen and Gibala, 2014), and growing evidence supports the benefits of HIIE for physical and mental health (Martland et al., 2020). Although a benefit of HIIE is its time efficiency, critics have argued that when incorporating a warm-up, and cooling down then HIIE can take as long as more traditional exercise (Hardcastle et al., 2014). Consequently, there has been increased interest in low volume high intensity interval exercise (LV-HIIE). LV-HIIE is defined as ≤ 10 min of intense exercise within a total exercise time of ≤ 30 min (including warm-up, rests, and cool down) (Gillen and Gibala, 2014). Some studies have suggested that the physiological adaptations induced by LV-HIIE are similar to or better compared to moderate intensity continuous exercise (Gibala et al., 2012; Weston et al., 2014).

With the growing interest and evidence for the health benefits of HIIE and LV-HIIE, there is continued debate regarding their relevance for public health (e.g., Biddle and Batterham, 2015). This debate centers primarily around the notion that due to the high-intensity nature of HIIE, it will result in an unpleasant affective response to the exercise (Ekkekakis, 2003), which in turn will reduce the likelihood of future engagement in HIIE (Rhodes and Kates, 2015). Indeed, recent review level evidence indicates that compared to continuous moderate intensity exercise, participants do experience HIIE as less pleasant at several points during exercise (Niven et al., 2020). However, there has been only limited consideration on how this affective response impacts future exercise behavior, and determinants of this behavior, such as self-efficacy and intentions (Stork et al., 2018).

Niven et al. (2020) noted a high degree of heterogeneity between studies and previously suggested that these mixed findings may be due to individual differences impacting different affective responses to exercise (Bradley et al., 2019). Indeed, Bradley et al. (2019) reported that self-reported very low tolerance of exercise intensity impacted negatively on affective responses to LV-HIIE, and intentions to repeat the activity. Contrary to this finding, Astorino et al. (2020) showed no difference in affective responses to reduced-exertion high-intensity interval training (REHIIT) between participants who scored above and below the average tolerance scores. However, the impact of exercise tolerance on affective responses is also evident in non-HIIE studies (e.g., Tempest and Parfitt, 2016). Given these contradictory findings, there is clearly a need for further research to consider the impact of exercise tolerance on affective responses to HIIE.

A further strategy to enhance the time efficiency of exercise is to exercise in the home, negating the need to travel to a facility or specific location. During the lockdown restrictions due to COVID-19, online exercise classes were recommended as one strategy to maintain physical activity levels (NHS Inform, 2021). Subsequently, there has been a growth in the availability of online classes, and their popularity has soared (Thompson, 2021). Although there is limited research on this topic, it is possible that in the “new normal” many will continue to exercise in this way. However, we know very little about how individuals respond to LV-HIIE in the home that can be used to support and encourage adherence. Indeed, we are unaware of any studies that have used exercise videos to administer LV-HIIE at home, and record responses, or even if research of this nature is feasible. Such studies are important to contribute to our understanding of how participants experience LV-HIIE in a more ecologically valid, non-laboratory setting.

Therefore, the aims and hypotheses of this study were to:

1) Demonstrate that it is possible to recruit people to a remotely delivered “at home” LV-HIIE intervention and conduct data collection in healthy adults. We hypothesized that it would be possible to recruit enough participants to conduct a sufficiently powered study.
2) Examine the influence of self-reported tolerance of the intensity of exercise on affective responses to remotely delivered “at home” LV-HIIE. We hypothesized that self-reported tolerance of the intensity of exercise would significantly influence affective responses, with higher self-reported tolerance individuals reporting significantly more positive affective responses than low self-reported tolerance individuals.
3) Assess the relationships between affective responses and subsequent self-efficacy and intentions to repeat remotely delivered “at home” LV-HIIE. We hypothesized that there would be significant positive relationships between affective responses and both self-efficacy and intentions to repeat.

## Materials and Methods

### Study Design

We employed a non-randomized parallel group design to assess the feasibility of recruitment and the between-group responses to a single bout of LV-HIIE. We used G^*^Power (version 3.1.9.4, Universitat Kiel, Germany) to calculate the required sample size to achieve a β = 0.90 to detect a large effect of tolerance of the intensity of exercise [F = 0.50; effect size from Bradley et al. (2019) was used] on affective valence from a factorial ANOVA with three independent groups. The required total sample size was 42.

### Target Population, Recruitment, and Screening

The target population was healthy active men and women (aged 18–45 for men and 18–55 for women due to different risk profiles) who were not elite athletes or training at a high level, were injury free, and met current UK Physical activity guidelines (≥150 mins of MVPA or ≥75 mins VPA, or combination of both (Department of Health Social Care, 2019). We recruited physically active rather than inactive individuals due to institutional ethical restrictions on the recruitment of physically inactive individuals to an unsupervised HIIE intervention.

Participant recruitment was through active methods (word of mouth, snowballing, social media announcements) with interested individuals giving permission to be contacted by the lead researcher by email. The Physical Activity Readiness Questionnaire (PAR-Q) and a Medical Screening Questionnaire (MSQ) were used to assess eligibility. These were sent by email to potential participants by the lead researcher and returned as forms assessed by the study team.

### Ethics and Safety

Ethical approval was given by the Moray House the School of Education and Sport Ethics Sub-Committee (Ref: PK20112020-1 25th January 2021). Informed consent forms were completed and electronically signed by participants prior to testing. A risk assessment was conducted and appropriate safety guidance was included in the video protocol. Guidance was also given when indicated by the PAR-Q or MSQ that did not exclude the participant but warranted extra safety considerations.

### Measures

A demographic questionnaire was used to assess age, sex, height, and weight. The 16-item Preference for and Tolerance of the Exercise Intensity Questionnaire (PRETIE-Q) (Ekkekakis et al., 2005) was used to assess participants' interpretation of interoceptive stimuli during exercise, to quantify their preference for and tolerance of the intensity of exercise. Each item comprises a five-point response scale (1 = I totally disagree to 5 = I strongly agree). The tolerance scale of the PRETIE-Q has been shown to predict affective responses, so the eight items on that scale were summed to provide a composite score of tolerance (possible range 8–40) used in this study. The PRETIE-Q has established test-retest reliability (Ekkekakis et al., 2005), and in the current study had an internal consistency (Cronbach's alpha) of 0.78.

The Borg CR-10 scale (Borg and Kaijser, 2006) assessed subjective ratings of perceived exertion (RPE), as a valid and reliable prediction tool. This was used as a manipulation check to confirm that the participants were experiencing high intensity exercise.

The Feeling Scale (FS) (Hardy and Rejeski, 1989) was used to assess affective valence (pleasure/displeasure) using a single item 10-point scale (ranging from −5 very bad to +5 very good). The Felt Arousal Scale (FAS) (Svebak and Murgatroyd, 1985) was used to assess perceived activation on a single item 6-point scale (ranging from 1 low arousal to 6 high arousal).

Task self-efficacy to regularly perform the HIIT protocol was assessed using a two-item measure (Jung et al., 2014; Bradley et al., 2019). The first item asked participants “How confident are you that you can perform one bout of exercise a week for the next four weeks that is just like the one you completed today?”. The second question was identical but referred to three bouts of exercise a week. Participants responded on a scale 0% (not at all) to 100% (extremely confident) using 10% increments. Intentions to engage in the HIIT protocol in the next month were assessed using a two-item measure (Jung et al., 2014; Bradley et al., 2019). Participants were asked to rate the extent to which they agreed on a 7-point scale ranging from 1 (very unlikely) to 7 (very likely) with the statement “I intend to engage in the type of exercise I performed today at least once per week during the next month”, and the same statement but with a frequency of at least three times per week.

### LV-HIIE Set-Up and Protocol

**Figure 1:**
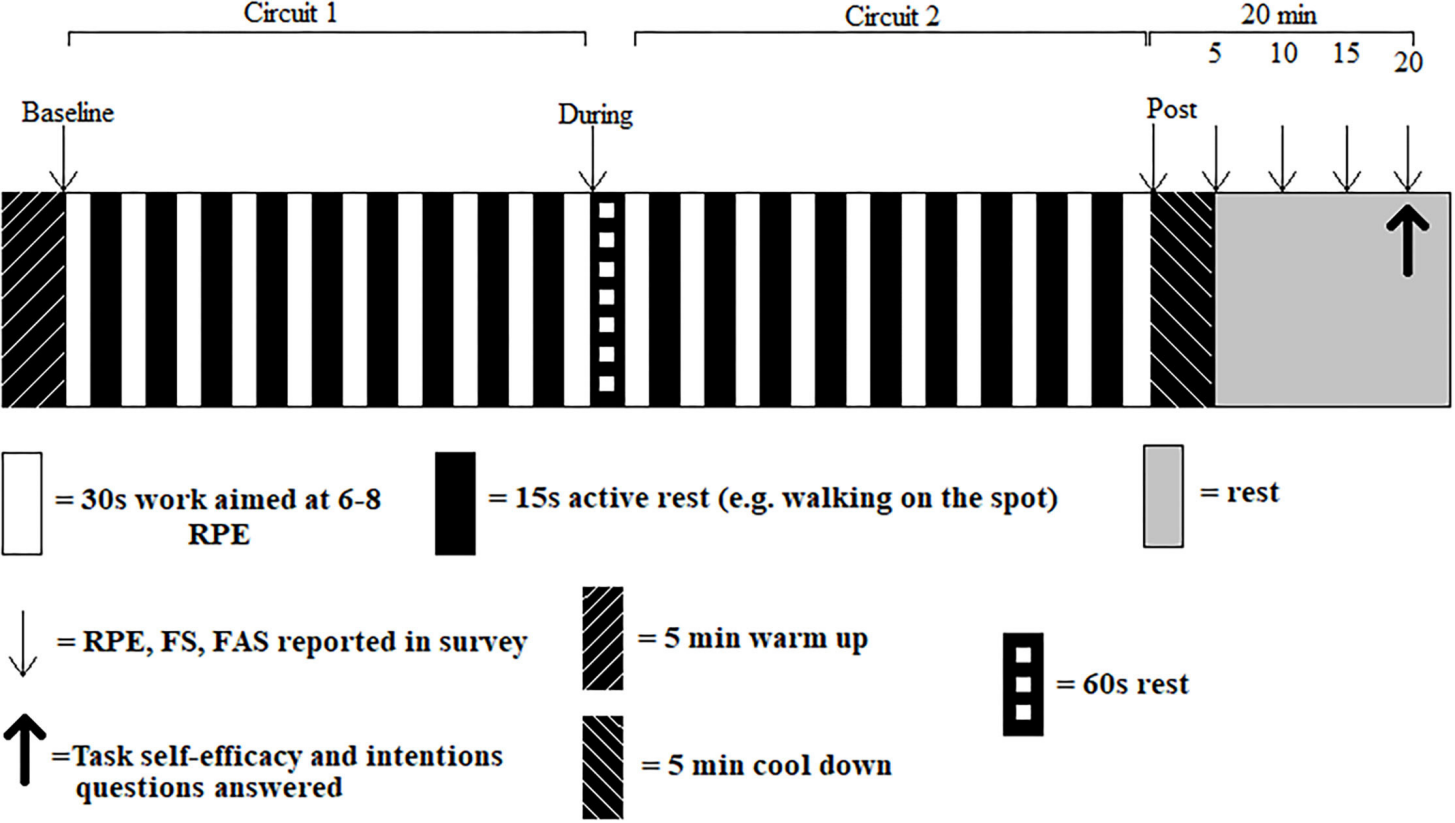
Exercise and data collection protocol. RPE, Rating of Perceived Exertion; FS, Feeling Scale; FAS, Felt Arousal Scale.

Included participants were sent an email link to the intervention video (privately accessed through YouTube). To reduce potential bias or other errors associated with self-reporting, the video included an initial standardized task explanation and familiarization of the measurement tools (both their use and the timing of the measurements). The 5 min warm-up included both static and dynamic stretches incorporating adapted movements that would be used in the LV-HIIE circuits. Then, two circuits of 10 exercises (30 s duration, interspersed with 15 s passive recovery) with a 60 s rest between circuits were completed (Table 1), followed by a 5 min cool-down. This protocol equates to 10 min of intense exercise within a total exercise duration of 25.5 min (Figure 1). Participants were asked to find a clear 2 × 1 m space in their homes to complete the protocol. External variables (e.g., temperature) were not standardized to simulate how people would choose to exercise at home, but participants were advised to be in an area of room temperature (~16–19°C) for their safety. Participants were instructed to refrain from alcohol and not complete PA ≤ 48 h before, to prevent compromising exercise perception (Marcora et al., 1985). Exercises were chosen based on previous similar research (Klika and Jordan, 2013). The exercises in the video were performed at a mean tempo of 130 beats per minute, in line with the tempo used in research investigating the effects of music on high-intensity interval exercise performance (Maddigan, 2013; Maddigan et al., 2019). Participants were instructed to maintain the same rhythm as the demonstrated exercise. This approach provided participants with the freedom to reduce the intensity of the exercise if required, but also gave them a target intensity for which to aim. This approach is more standardized than other at-home video-based exercise interventions, which simply played music in the videos in an attempt to increase exercise intensity (McDonough et al., 2022).

**Table 1:** Activities in the exercise protocol.

**1**	**2**	**3**	**4**	**5**	**6**	**7**	**8**	**9**	**10**
High knees at running pace	Plank walk outs	Jump lunges	Squat with upward diagonal crunch knee to elbow (alternating sides)	Star jumps	Squat jumps	Lunge to crunch (left)	Lunge to crunch (right)	Burpees	High knees at running pace
						During the rising phase the following (behind body) leg is raised			

### Data Collection

Data collection took place in Feb-Mar 2021. Participant study data were self-reported using survey software Qualtrics (Qualtrics, Utah). A link to the survey was sent in the same email as the intervention video.

Exercise tolerance data were reported before the exercise protocol. Anthropometric data were self-reported and used to calculate BMI (Olfert et al., 2018). Guidance was included in the video, provided by a Level 3 accredited anthropometrist.

RPE and affective measures were self-reported before, during, and post LV-HIIE, within and following the cool down to account for affective rebound (Ekkekakis et al., 2005). This was done by asking the participant to simultaneously view the survey and the protocol video in a split screen desktop view on their personal device. Reporting in the 60s rest between circuits and immediately post-circuit two was advised to be in the first 10s of rest before the affective rebound (Roloff et al., 2020).

Cool down measures were reported at 5-min intervals across 20-min (Figure 1). For statistical analysis and presentation, the average of these four values was calculated. At 20-min post-protocol, participants reported the final affective measures and answered task self-efficacy and intentions questions (Jung et al., 2014; Bradley et al., 2019).

### Statistical Analyses

Analyses were conducted using IBM SPSS Statistics 24 for Windows (IBM Corp., Chicago, IL) with an alpha level of *p* ≤ 0.05 applied. Data normality was assessed using the Shapiro-Wilk test. Participant characteristics were compared between tolerance groups using a one-way independent groups analysis of variance (ANOVA). Tolerance groups were based on tertiles, but participants with the same score were placed in the same group giving uneven group sizes. Rating of perceived exertion, affective valence, and felt arousal were analyzed using a two-way (tolerance × time) mixed-methods ANOVA, with Bonferroni correction applied to all *post hoc* pairwise comparisons. Pearson product moment correlations analyzed the relationships between affective valence at each time-point and intention to repeat and self-efficacy data. The magnitude of the correlations was classified as negligible (±0 to 0.30, low (±0.30 to 0.50), moderate (±0.50 to 0.70), high (±0.70 to 0.90), and very high (±0.90 to 1.00) (Mukaka, 2012).

Effect size (ES) estimates based on sample data overestimate the true population effect. Therefore, we applied a correction to all ES to mitigate this bias, as described by Cumming (2011). All ES in this study is presented as unbiased d (d_*unb*_), in line with the convention recommended by Cumming (2011). We calculated 95%CL for the ES using the procedure described by Algina and Keselman (Algina and Keselman, 2003). The magnitude of the ES was defined as trivial (d <0.2), small (d ≥ 0.2, <0.5), medium (d ≥ 0.5, <0.8), and large (d ≥ 0.8) (Cohen, 1992), expressed in units of standard deviation.

## Results

### Recruitment, Delivery, and Data Collection for a Remote “At Home” LV-HIIE Intervention

Of the 65 individuals recruited, 50 participants passed screening for inclusion in the study. Reasons for exclusion included changed mind (*n* = 6), did not pass PAR-Q (*n* = 1), elite athlete (*n* = 1), injured (*n* = 3), PA level too low (*n* = 2), no access to BMI measures (*n* = 1), and already regular HIIE participant i.e., >5 times per week (*n* = 1).

Of the 50 recruited participants, 41 (82%) ultimately completed the study protocol and provided full data. Two participants withdrew due to injury, three for undisclosed reasons and four only partially finished the survey and were discounted.

Participant demographics for the final sample of *n* = 41 are shown in Table 2. There were more women (61%) than men, and the participants were generally young adults [mean age 21.3 years with a low standard deviation (1.0)], and the mean BMI (23.0) was in the healthy range.

**Table 2:** Participant demographics.

	**All (*n* =41)**	**Lowest (*n =* 14)**	**Middle (*n =* 15)**	**Highest (*n =* 12)**	**Between group differences (*p*)**
Age (years)	21.3 (1.0)	21.5 (0.7)	21.3 (1.0)	21.1 (1.2)	0.450
Gender	25 F, 16 M	11 F, 3 M	9 F, 6 M	5 F, 7 M	0.621
Height (cm)	174.5 (9.0)	170.6 (7.8)	173.3 (7.4)	180.6 (10.0)*	0.015
Body mass (kg)	70.1 (11.7)	66.4 (8.7)	67.6 (13.0)	77.6 (10.9)*	0.028
BMI (kg.m^−2^)	23.0 (2.9)	22.8 (2.7)	22.4 (3.6)	23.7 (2.3)	0.501
**Tolerance**					
Range	18–37	18–24	25–29	30–37	
Median	27.0	23	27	33	
Mean	26.9	21.9	27.2	32.5	

*Significantly greater than low tolerance, p < 0.05.

Participant recruitment took 6 weeks and data collection lasted 5 weeks. The main issues included difficulty recruiting participants without direct face-to-face contact and minimizing risks during data collection by educating participants about potential hazards and how to prevent them from occurring.

#### Manipulation Check

**Figure 2:**
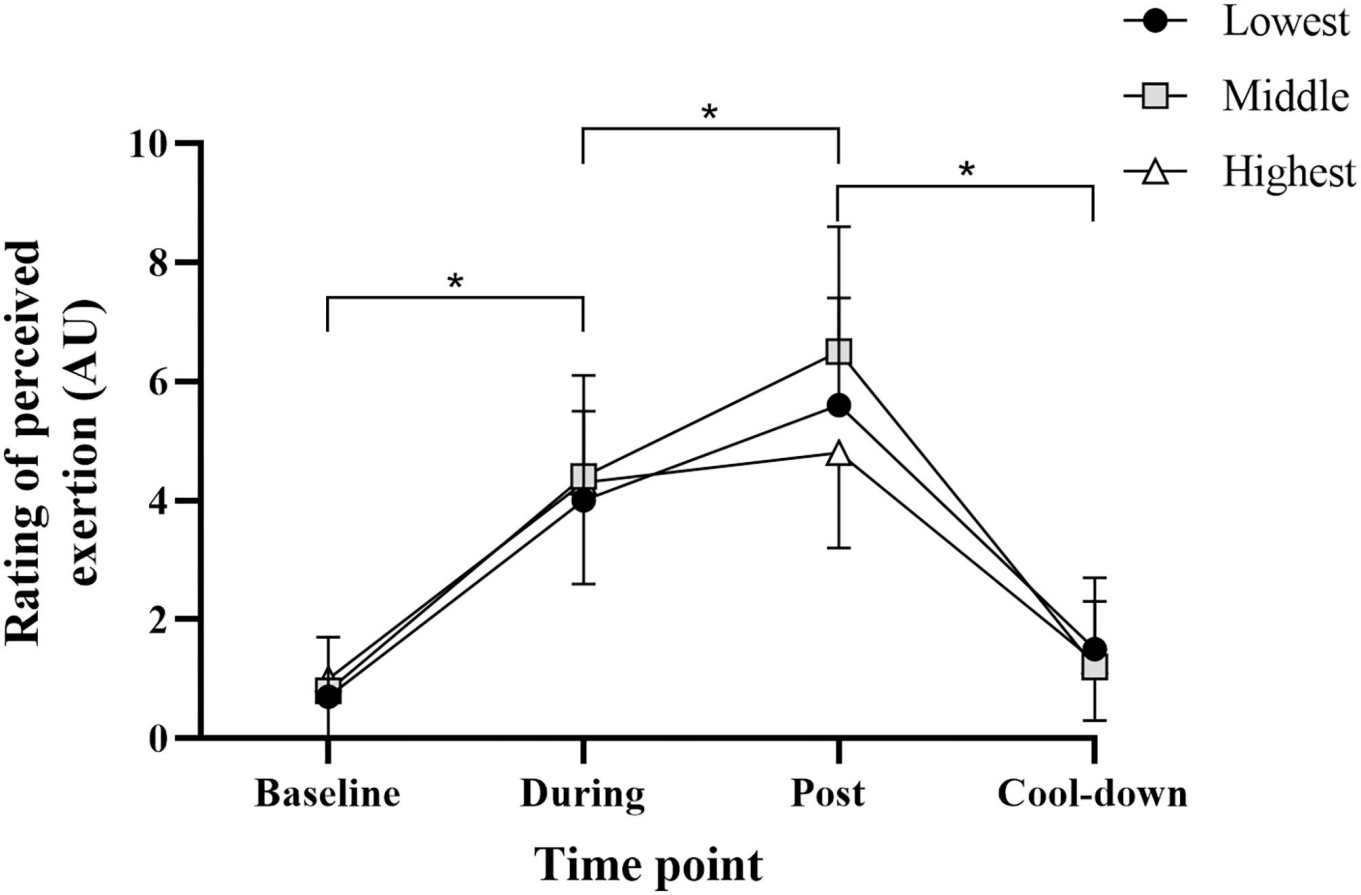
Rating of perceived exertion for all groups at each time point. Data are mean (SD). *Significant pairwise comparison (*p* < 0.001).

There was no significant main effect of tolerance (*F*_2, 38_ = 0.598, *p* = 0.56) or tolerance × time interaction (*F*_6, 114_ = 1.560, *p* = 0.17) for RPE (Figure 2). There was a significant main effect of time (*F*_3, 114_ = 117.85, *p* < 0.001). Rating of perceived exertion increased significantly from baseline to during and during to post, and decreased significantly from post to cool down (all comparisons *p* < 0.001, d_*unb*_ = 0.89–2.84). These data confirm similar perceptions of task difficulty were present across tolerance groups.

### Influence of Self-Reported Tolerance of the Intensity of Exercise on Affective Responses to Remotely Delivered “At Home” LV-HIIE

**Figure 3:**
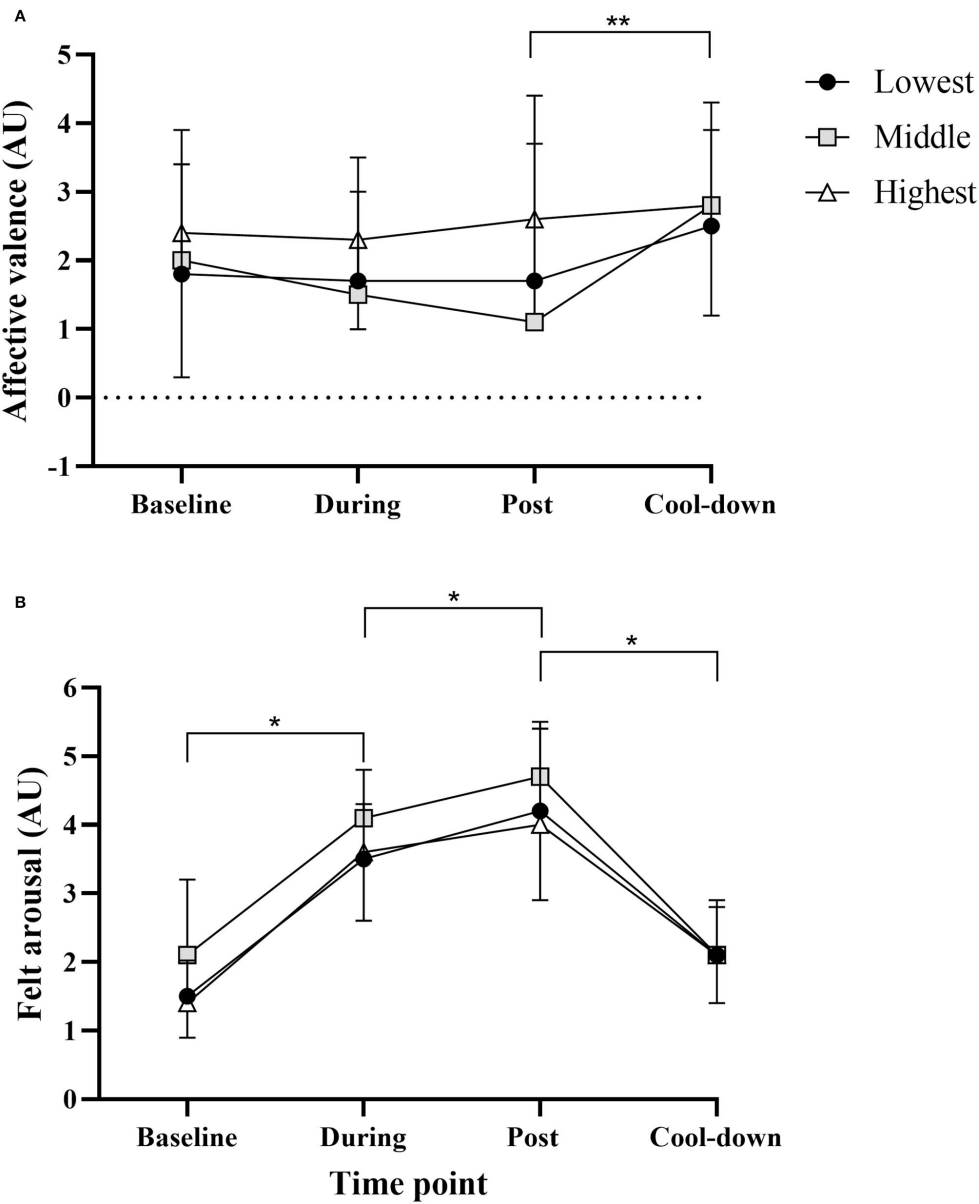
Affective valence **(A)** and felt arousal **(B)** data for all groups at each time point. Data are mean (SD). *Significant pairwise comparison (*p* < 0.001); **Significant main effect of time (*p* = 0.04).

Affective valence and felt arousal data by self-reported exercise tolerance are shown in Figure 3.

For affective valence (Figure 3A), there was no significant main effect of tolerance (*F*_2, 38_ = 1.047, *p* = 0.36) or time × tolerance interaction (*F*_6, 114_ = 0.62, *p* = 0.72). There was a significant main effect of time (*F*_3, 114_ = 2.79, *p* = 0.04). Affective valence increased significantly from post to cool down (p = 0.018, d_*unb*_ = 0.47). This suggests a degree of “affective rebound” at cool-down.

For felt arousal (Figure 3B), there was no significant main effect of tolerance (*F*_2, 38_ = 3.03, *p* = 0.06) or tolerance x time interaction (*F*_6, 114_ = 0.99, *p* = 0.44). There was a significant main effect of time (*F*_3, 114_ = 116.26, *p* < 0.001). Felt arousal increased significantly from baseline to during and during to post, and decreased significantly from post to cool down (all comparisons *p* < 0.001, d_*unb*_ = 0.45–1.52).

**Figure 4:**
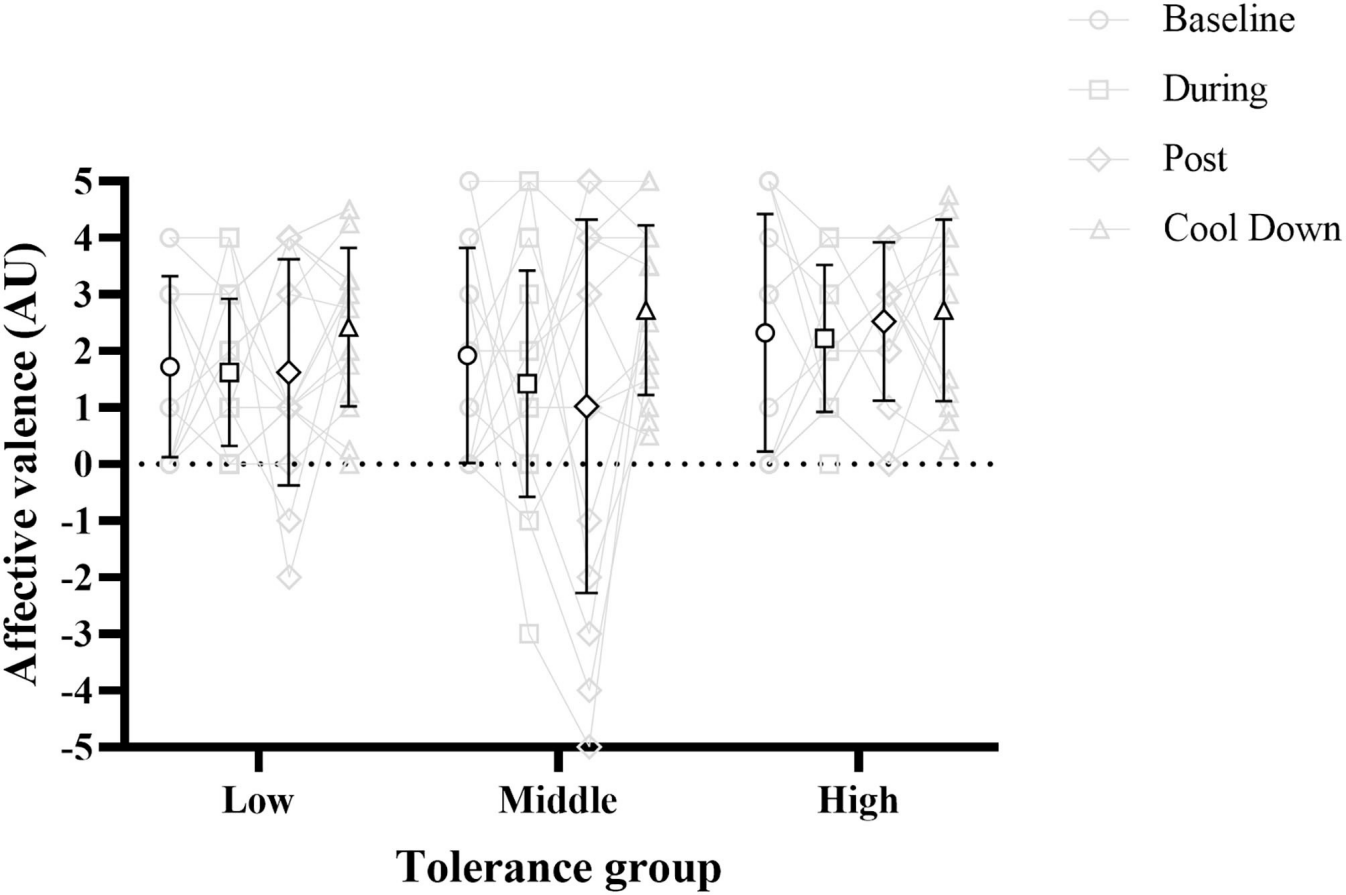
Affective valence at each time point for all tolerance groups. Symbols with black outline are mean, with error bars representing standard deviation. Data in gray is individual participant data. Gray lines represent a single participant across each time point.

Of note is the high variation in affective valence and felt arousal at each time-point, as shown by the standard deviations in Figure 3. Figure 4 presents the affective valence data in more detail, showing the individual participant data at each time point. This shows that there is a wide range of affective valence with scores at every point on the scale (−5 to +5). A large proportion of scores however are in the pleasant part of the scale (+1 to +5). The low and high tolerance groups look similar, while the middle tolerance group has a number of values in the unpleasant range (−1 to −5) both during and immediately post exercise, which has returned to the pleasant range at cool-down.

**Figure 5:**
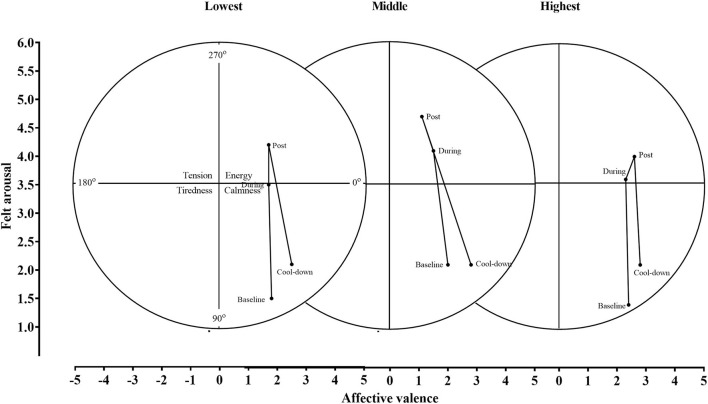
The circumplex model applied to all groups.

The patterns of the circumplex model for each group are shown in Figure 5. The patterns were similar across groups, with low activation and positive affect (sense of calmness) prior to exercise and during the cool-down period, and high activation and positive affect (sense of energy) during exercise. At no point in any group did participants at the group level experience high activation and negative affect (tension) or low activation and negative affect (tiredness).

### Relationships Between Affective Responses and Subsequent Intentions and Self-Efficacy to Repeat Remotely Delivered “At Home” LV-HIIE

Correlation analyses between affective valence at each time point and subsequent intention and self-efficacy data are shown in Table 3. There were no statistically significant correlations between affective valence and intention or self-efficacy data at any time point. All correlations were positive except for correlations between cool down affective valence and self-efficacy, and the magnitude of all correlations was negligible to low.

**Table 3:** Correlation analyses between affective valence at each time point and intention and self-efficacy data.

**Outcome measure**				
**Affective valence**	**Intention to repeat once per week**	**Intention to repeat three times per week**	**Self-efficacy once per week**	**Self-efficacy three times per week**
Pre-exercise	0.13 (0.41)	0.30 (0.06)	0.0 (0.99)	0.04 (0.79)
During exercise	0.00 (0.98)	0.01 (0.94)	0.09 (0.57)	0.02 (0.89)
Post-exercise	0.31 (0.05)	0.25 (0.12)	0.11 (0.51)	0.10 (0.53)
Cool down	0.29 (0.06)	0.15 (0.36)	−0.09 (0.56)	−0.13 (0.42)

*Data presented as r (p value)*.

## Discussion

There is now a substantial evidence base for the health benefits of HIIE (Martland et al., 2020) and LV-HIIE (Weston et al., 2014), but there is a need to better understand exercisers' affective experience of HIIE to determine its value for public health. A better understanding of affective responses and moderators of these responses will enable better targeting of physical activity recommendations. This research has made a unique contribution to this understanding by examining affective responses to LV-HIIE “at home”. Although studies have explored this previously in laboratory settings (e.g., Niven et al., 2018; Campbell and Phillips, 2020), there is little evidence regarding responses in a more ecologically valid setting, and indeed if such studies can be undertaken. Overall, it was shown to be possible to recruit to and deliver a remote “at home” LV-HIIE single bout exercise intervention and collect research data. Indeed, the recruited sample was larger than many laboratory studies, and provided sufficient power to detect the expected effect based on previous laboratory research. Therefore, our first hypothesis was accepted. Contrary to our second hypothesis the findings indicated that there was no effect of exercise tolerance on affective responses, although there was evident variation in responses. Finally, affective valence was not related to future intentions or self-efficacy to engage in the exercise again, and thus the third hypothesis was also rejected.

Although recently the popularity of home-based exercise has increased considerably (Thompson, 2021), there is limited understanding of participants' responses to exercise in this setting. In our study, we showed that it was possible to recruit participants and engage them in an LV-HIIE session that was standardized, as indicated by no differences in RPE across groups. It was also possible to collect data on affective (and psychological) outcomes before, during, and after the protocol. Taken together, these findings on recruitment and data collection indicate that research can be conducted out of the laboratory in a more ecologically valid way to study the effects of at-home LV-HIIE.

Previous research has indicated that affective responses to LV-HIIE can be influenced by self-reported exercise tolerance (Bradley et al., 2019). However, the data from this study on affective responses, including valence and arousal, did not reveal any clear patterns in the exercise tolerance group and did not support the findings from this previous study or other non-HIIE studies (e.g., Tempest and Parfitt, 2016). However, this finding was consistent with previous REHIIT research that also showed no influence of exercise tolerance on affective responses (Astorino et al., 2020). The data did however indicate that there was wide individual variation in affective responses to LV-HIIE, which is consistent with other research (Olney et al., 2018) and reinforces the value of identifying moderators of these responses. Previous research has reported that physical activity status and RPE response (Farias-Junior et al., 2020), baseline effect, blood lactate concentration, post-exercise enjoyment (Astorino and Vella, 2018), and low fitness levels (Astorino et al., 2020) are related to affective responses to HIIE. Future research is needed to further consider the role of these previously identified variables in moderating the affective responses to LV-HIIE at home in order to optimize the experience.

It was notable that there was limited evidence of unpleasant affective response within and across the groups, with only a handful of participants reporting negative affect, and limited reduction in affect across the protocol. This is encouraging, indicating that home-based LV-HIIE does not universally lead to negative affect. Nevertheless, this finding is contrary to previous laboratory research that has shown reductions in affect during HIIE (e.g., Astorino and Vella, 2018). This limited decrease in affect in the current study may be due to the home-based nature of the activity, although this needs to be explored by research comparing home-based HIIE with other environments, such as a gym and exercise science laboratory. Affective responses to HIIE are significantly influenced by physical activity status, with physically active participants showing significantly more positive affect during an HIIE protocol comprised of 10 × 60 s treadmill running interspersed with 60-s recoveries (Frazao et al., 2016). Therefore, the physically active status of our participants may account for the limited evidence of unpleasant effect. Future research is needed to understand how these findings compare with non-LV-HIIE exercise in the home environment and in less active and/or healthy participants Nevertheless, this finding showing limited negative effect suggests home-based LV-HIIE may be a palatable form of health-enhancing activity that is also convenient and time efficient thus addressing perceived lack of time, which is a commonly cited barrier to physical activity.

The affective responses to HIIE have been a focus of research attention because it is expected that these responses will influence future behavior (Rhodes and Kates, 2015); however, only limited research has considered this issue. In this study, we found a limited relationship between affective valence at different stages of exercise and the cognitive antecedents of intention to, and self-efficacy for future engagement in LV-HIIE. This finding is consistent with a previous study (Stork et al., 2018) which reported that the in-task affect did not predict the future behavior of high-intensity training. Although the findings in this study may be limited by sample size, as noted elsewhere (Niven et al., 2020) or the lack of variation in participants' responses (e.g., almost all participants rated intention at 6 or 7), future research is clearly needed to further unpick the affect-cognitive antecedent-behavior relationship in HIIE to build on what is known from continuous exercise (Rhodes and Kates, 2015).

### Strengths and Limitations

A strength of this study is its novelty of successfully delivering and studying LV-HIIE in an ecologically valid home environment, with a relatively large sample compared to many laboratory studies (Hall et al., 2020). However, the recruited sample represents a narrow demographic, namely young adults in a healthy BMI range, which limits the generalizability of the findings. Future research with other demographic groups, such as older ages and overweight groups, would be valuable. A limitation of maximizing ecological validity is that certain outcomes would normally be collected in a laboratory setting that could not be tested in this protocol, including heart rate, baseline VO_2_max, and ventilatory threshold or expertly measured anthropometric measures. Finally, due to the nature of the exercise, it was not possible to capture affective responses during the activity as it would have disrupted the execution of the LV-HIIE circuit. Instead, participants reported during the first 10 s of rest to minimize the effect rebound (Roloff et al., 2020); however, it is noted that the rebound effect would not have been fully eliminated.

## Conclusion

This study shows it is possible to recruit young people of healthy weight status to an “at home” single bout LV-HIIE exercise protocol study. It is also possible to deliver the protocol stimulating appropriate exercise intensities and to collect a range of psychological data.

Although there was a large degree of variation in affective responses to LV-HIIE, contrary to previous research, exercise tolerance within this sample did not pattern the experience or perception of LV-HIIE. This finding highlights the need for further research to better understand moderators of the variation in affective responses to LV-HIIE.

Affective responses had a limited relationship with cognitive antecedents of future behavior, potentially suggesting that other factors may be more important to predict future engagement in LV-HIIE. Assessing self-reported tolerance of intensity of exercise may not appropriately identify whether or not LV-HIIE will be appropriate for an individual, at least in this population.

Future research that addresses these points will provide better information about the health promotion approaches, and help identify for whom LV-HIIE is most suitable.

## Data Availability Statement

The raw data supporting the conclusions of this article will be made available by the authors, without undue reservation.

## Ethics Statement

The studies involving human participants were reviewed and approved by University of Edinburgh, Moray House School of Education and Sport Ethics Sub-Committee. The patients/participants provided their written informed consent to participate in this study.

## Author Contributions

IH, PK, AN, and SP established the research questions, designed the methodology, carried out data analysis, and co-wrote the manuscript. IH prepared all data collection resources and led pilot work and data collection. All authors contributed to the article and approved the submitted version.

## Conflict of Interest

The authors declare that the research was conducted in the absence of any commercial or financial relationships that could be construed as a potential conflict of interest.

## Publisher's Note

All claims expressed in this article are solely those of the authors and do not necessarily represent those of their affiliated organizations, or those of the publisher, the editors and the reviewers. Any product that may be evaluated in this article, or claim that may be made by its manufacturer, is not guaranteed or endorsed by the publisher.

## References

[B1] Algina J. Keselman H. J. (2003). Approximate confidence intervals for effect sizes. Educ. Psychol. Meas. 63, 537–553. 10.1177/0013164403256358

[B2] Astorino T. A. Clausen R. Marroquin J. Arthur B. Stiles K. (2020). Similar perceptual responses to reduced exertion high intensity interval training (REHIT) in adults differing in cardiorespiratory fitness. Physiol. Behav. 213, 112687. 10.1016/j.physbeh.2019.112687 31622613

[B3] Astorino T. A. Vella C. A. (2018). Predictors of change in affect in response to high intensity interval exercise (HIIE) and sprint interval exercise (SIE). Physiol. Behav. 196, 211–217. 10.1016/j.physbeh.2018.08.017 30170171

[B4] Biddle S. Batterham A. (2015). High-intensity interval exercise training for public health: a big HIT or shall we HIT it on the head? Int. J. Behav. Nutr. Phys. Act. 12, 1–8. 10.1186/s12966-015-0254-9 26187579 PMC4506613

[B5] Biddle S. Mutrie N. Gorely T. Faulkner G. (2021). Psychology of physical activity: Determinants, well-being and interventions. New York: Routledge. (2021). 10.4324/9781003127420

[B6] Borg E. Kaijser L. (2006). A comparison between three rating scales for perceived exertion and two different work tests. Scand J. Med. Sci. Spor. 16, 57–69. 10.1111/j.1600-0838.2005.00448.x 16430682

[B7] Bradley C. Niven A. Phillips S. M. (2019). Self-reported tolerance of the intensity of exercise influences affective responses to and intentions to engage with high-intensity interval exercise. J. Sports Sci. 37, 1472–1480. 10.1080/02640414.2019.1570590 30694110

[B8] Campbell J. Phillips S. M. (2020). The effects of two weeks low-volume self-regulated high-intensity interval training on cardiorespiratory fitness, exercise enjoyment, and intentions to repeat. J. Hum. Sport Exerc. 16, 411–23. 10.14198/jhse.2021.162.15

[B9] Cohen J. (1992). A power primer. Psychol. Bull. 112, 155–159. 10.1037/0033-2909.112.1.155 19565683

[B10] Cumming G. (2011). Understanding The New Statistics: Effect Sizes, Confidence Intervals, and Meta-Analysis (1st ed.). Routledge.

[B11] Department of Health and Social Care (2019). UK Chief Medical Officers' Physical Activity Guidelines. London, UK.

[B12] Ekkekakis P. (2003). Pleasure and displeasure from the body: perspectives from exercise. Cogn. Emot. 17, 213–239. 10.1080/02699930302292 29715726

[B13] Ekkekakis P. Hall E. E. Petruzzello S. J. (2005). Some like it vigorous: Measuring individual differences in the preference for and tolerance of exercise intensity. J. Sport Exerc. Psychol. 27, 350–374. 10.1123/jsep.27.3.350

[B14] Farias-Junior L. F. Browne R. A. V. Astorino T. A. Costa E. C. (2020). Physical activity level and perceived exertion predict in-task affective valence to low-volume high-intensity interval exercise in adult males. Physiol. Behav. 224, 112960. 10.1016/j.physbeh.2020.112960 32659496

[B15] Frazao D. T. de Farias Dantas, L. F. Krinski T. C. Elsangedy K. Prestes H. M. J.. (2016). Feeling of pleasure to high-intensity interval exercise is dependent of the number of work bouts and physical activity status. PLoS ONE. 11, e0153986. 10.1371/journal.pone.0153986 27077908 PMC4831771

[B16] Gibala M. J. Little J. P. MacDonald M. J Hawley J. A. (2012). MacDonald MJ, Hawley JA. Physiological adaptations to low-volume, high-intensity interval training in health and disease. J. Physiol. 590, 1077–1084. 10.1113/jphysiol.2011.224725 22289907 PMC3381816

[B17] Gillen J. B. Gibala M. J. (2014). Is high-intensity interval training a time-efficient exercise strategy to improve health and fitness? Appl. Physiol. Nutr. Metab. 39, 409–412. 10.1139/apnm-2013-0187 24552392

[B18] Guthold R. Stevens G. A. Riley L. M. Bull F. C. (2018). Worldwide trends in insufficient physical activity from 2001 to 2016: a pooled analysis of 358 population-based surveys with 1and#xb7;9 million participants. Lancet Global Health. 6, e1077–e86. 10.1016/S2214-109X(18)30357-7 30193830

[B19] Hall A. Aspe R. Craig T. Kavaliauskas M. Babraj J. Swinton P. (2020). The effects of sprint interval training on physical performance: a systematic review and meta-analysis. Scand. J. Med. Sci. Sports. 23:e341–52. 10.31236/osf.io/nphw2 36165995

[B20] Hallal P. C. Andersen L. B. Bull F. C. Guthold R. Haskell W. Ekelund U. (2012). Global physical activity levels: surveillance progress, pitfalls, and prospects. Lancet. 380, 247–257. 10.1016/S0140-6736(12)60646-1 22818937

[B21] Hardcastle S. J. Ray H. Beale L. Hagger M. S. (2014). Why sprint interval training is inappropriate for a largely sedentary population. Front. Psychol. 5, 1505. 10.3389/fpsyg.2014.01505 25566166 PMC4274872

[B22] Hardy C. J. Rejeski W. J. (1989). Not what, but how one feels - the measurement of affect during exercise. J. Sport Exerc. Psychol. 11, 304–317. 10.1123/jsep.11.3.304

[B23] Jung M. E. Bourne J. E. Little J. P. (2014). Where does HIT fit? An examination of the affective response to high-intensity intervals in comparison to continuous moderate- and continuous vigorous-intensity exercise in the exercise intensity-affect continuum. PLoS ONE. 9, e114541. 10.1371/journal.pone.0114541 25486273 PMC4259348

[B24] Klika B. Jordan C. (2013). High-intensity circuit training using body weight: Maximum Results With Minimal Investment. ACSMs Health Fit J. 17. 10.1249/FIT.0b013e31828cb1e8

[B25] Lear S. A. Hu W. Rangarajan S. Gasevic D. Leong D. Iqbal R. (2017). The effect of physical activity on mortality and cardiovascular disease in 130 000 people from 17 high-income, middle-income, and low-income countries: the PURE study. Lancet. 390, 2643–2654. 10.1016/S0140-6736(17)31634-3 28943267

[B26] Maddigan M. E. (2013). The Effect of High Tempo Music as an External Stimulus During High Intensity Exercise. Memorial University of Newfoundland.

[B27] Maddigan M. E. Sullivan K. M. Halperin I. Basset F. A. Behm D. G. (2019). High tempo music prolongs high intensity exercise. PeerJ. 6, e6164. 10.7717/peerj.6164 30643679 PMC6329333

[B28] Marcora S. M. Staiano W. Manning V. (1985). Mental fatigue impairs physical performance in humans. J. Appl. Physiol. 106, 857–864. 10.1152/japplphysiol.91324.2008 19131473

[B29] Martland R. Mondelli V. Gaughran F. Stubbs B. (2020). Can high-intensity interval training improve physical and mental health outcomes? A meta-review of 33 systematic reviews across the lifespan. J. Sports Sci. 38, 430–469. 10.1080/02640414.2019.1706829 31889469

[B30] McDonough D. J. Helgeson M. A. Liu W. Gao Z. (2022). Effects of a remote, YouTube-delivered exercise intervention on young adults' physical activity, sedentary behavior, and sleep during the COVID-19 pandemic: Randomized controlled trial. J Sport Health Sci. 11, 145–156. 10.1016/j.jshs.2021.07.009 34314877 PMC8487769

[B31] Mukaka M. M. (2012). Statistics corner: a guide to appropriate use of correlation coefficient in medical research. Malawi Med. J. 24, 69–71. 23638278 PMC3576830

[B32] NHS Inform. (2021). Coronavirus (COVID-19): Physical Activity 2020. Available online at: https://www.nhsinform.scot/illnesses-and-conditions/infections-and-poisoning/coronavirus-covid-19/healthy-living/coronavirus-covid-19-physical-activity.

[B33] Niven A. Laird Y. Saunders D. H. Phillips S. M. (2020). A systematic review and meta-analysis of affective responses to acute high intensity interval exercise compared with continuous moderate- and high-intensity exercise. Health Psychol. Rev. 2020, 1–113. Available online at: 10.1080/17437199.2020.1728564 32067574

[B34] Niven A. Thow J. Holroyd J. Turner A. P. Phillips S. M. (2018). Comparison of affective responses during and after low volume high-intensity interval exercise, continuous moderate- and continuous high-intensity exercise in active, untrained, healthy males. J. Sports Sci. 36, 1993–2001. 10.1080/02640414.2018.1430984 29376774

[B35] Olfert M. D. Barr M. L. Charlier C. M. Famodu O. A. Zhou W. Mathews A. E. (2018). Self-Reported vs. Measured Height, Weight, and BMI in Young Adults. Int. J. Environ. Res. Public Health. 15, 2216. 10.3390/ijerph15102216 30314261 PMC6210375

[B36] Olney N. Wertz T. LaPorta Z. Mora A. Serbas J. Astorino T. A. (2018). Comparison of acute physiological and psychological responses between moderate-intensity continuous exercise and three regimes of high-intensity interval training. J. Strength Cond. Res. 32, 2130–2138. 10.1519/JSC.0000000000002154 28737586

[B37] Rhodes R. E. Kates A. (2015). Can the affective response to exercise predict future motives and physical activity behavior? A systematic review of published evidence. Ann. Behav. Med. 49, 715–731. 10.1007/s12160-015-9704-5 25921307

[B38] Roloff Z. A. Dicks N. D. Krynski L. M. Hartman M. E. Ekkekakis P. (2020). Pettitt R,. Ratings of affective valence closely track changes in oxygen uptake: application to high-intensity interval exercise. Perform. Enhanc. Health. 7, 100158. 10.1016/j.peh.2020.100158

[B39] Stork M. J. Gibala M. J. Martin Ginis K. A. (2018). Psychological and behavioral responses to interval and continuous exercise. Med. Sci. Sports Exerc. 50, 2110–2121. 10.1249/MSS.0000000000001671 29771824

[B40] Svebak S. Murgatroyd S. (1985). Metamotivational dominance - a multimethod validation of reversal theory constructs. J. Pers. Soc. Psychol. 48, 107–116. 10.1037/0022-3514.48.1.10718835411

[B41] Tempest G. Parfitt G. (2016). Self-reported tolerance influences prefrontal cortex hemodynamics and affective responses. Cogn. Affect. Behav. Neurosci. 16, 63–71. 10.3758/s13415-015-0374-3 26337703

[B42] Thompson W. R. (2021). Worldwide survey of fitness trends for 2021. ACSMs Health Fit J. 25, 10–19. 10.1249/FIT.000000000000063134444845

[B43] Weston M. Taylor K. L. Batterham A. M. Hopkins W. G. (2014). Effects of low-volume high-intensity interval training (HIT) on fitness in adults: a meta-analysis of controlled and non-controlled trials. Sports Med. 44, 1005–1017. 10.1007/s40279-014-0180-z 24743927 PMC4072920

